# Geometric Assortative Growth Model for Small-World Networks

**DOI:** 10.1155/2014/759391

**Published:** 2014-01-23

**Authors:** Yilun Shang

**Affiliations:** ^1^Singapore University of Technology and Design, Singapore 138682; ^2^Institute for Cyber Security, University of Texas at San Antonio, TX 78249, USA

## Abstract

It has been shown that both humanly constructed and natural networks are often characterized by small-world phenomenon and assortative mixing. In this paper, we propose a geometrically growing model for small-world networks. The model displays both tunable small-world phenomenon and tunable assortativity. We obtain analytical solutions of relevant topological properties such as order, size, degree distribution, degree correlation, clustering, transitivity, and diameter. It is also worth noting that the model can be viewed as a generalization for an iterative construction of Farey graphs.

## 1. Introduction

Recent studies of networked systems have led to the construction of models to explore their relevant properties, as one of the fundamental steps to understanding real-world phenomena of many kinds. Among them, small-world effect and network transitivity (or clustering) have attracted great research attention [1, 2]. Many real-life systems, such as social networks, food webs, World Wide Web, and airport networks, show both a high level of local clustering, similar to a regular lattice, and a relatively small average distance or diameter, namely, small-world effect, similar to a random graph. Networks with these two distinguishing characteristics are often said to be small-world networks.

The first and seminal model of small-world network is the Watts-Strogatz rewiring model [1], which induced an avalanche of works on studying small-world effect of complex networks and setting up variant models to expound the mechanism of small-world phenomenon. A variety of models of small-world networks have been studied, including stochastic ones modeled by adding randomness to regular graphs [1–7] and deterministic ones by making use of graph construction on some specific graphs such as planar lattices and Cayley graphs [8–11].

In this paper, we study a geometric growth model *G*(*m*, *t*) for small-world networks controlled by a tunable parameter *m*. Our model is constructed in a deterministic and recursive fashion. At each step, a multiple of *m* vertices will be added into the network as per some simple geometric structure. Compared with probabilistic methods, our model has some remarkable features. First, the model evolves through time which mimics the network growth in many real-world systems. Second, the simple generation method yields to analytical treatment of relevant topological properties include order, size, degree distribution and correlations, clustering, transitivity, and diameter. Finally, the model shows assortative mixing on the degrees, which is observed in varied social networks and has profound implications for network resilience [12, 13]. Many of the important properties studied in this paper (as mentioned above) are tunable by adjusting the parameter *m* in the model. For example, we show that the level of assortativity increases with *m* in terms of Pearson correlation coefficient, while clustering as well as transitivity coefficients decrease with *m*. This gives interesting characterization of a family of social network models, since both properties (i.e., assortative mixing and local clustering) are prevalent in social networks. Moreover, although the diameter always grows proportionally to the logarithm of the number of nodes in the network (hence displaying the small-world effect), it is shown to have distinct values for *m* = 1 and *m* > 1.  Table 1 summarizes the main contributions.

Here, we should mention prior work that is conceptually or spiritually relevant. The *m* = 1 case of our model was proposed in [14] as an alternative construction for Farey graphs. Farey graphs have many interesting properties such as minimally 3-colorable, uniquely Hamiltonian, maximally outerplanar and perfect; see, for example, [15]. Random constructions of Farey graph were explored in [16, 17], where an edge is removed with some probability *q* and *q*(*t*) at each step, respectively. Also for a different purpose, Dorogovtsev et al. [18] used a similar deterministic iteration process to generate pseudofractal scale-free networks (see also [19]). They have relevant but distinct properties with respect to our model.

The rest of the paper is organized as follows. In Section 2, we present our growth model for small-world networks. We report the structure properties of the model in Section 3. We conclude the paper in Section 4 with open problems.

## 2. The Network Model *G*(*m*, *t*)

**Figure 1:**
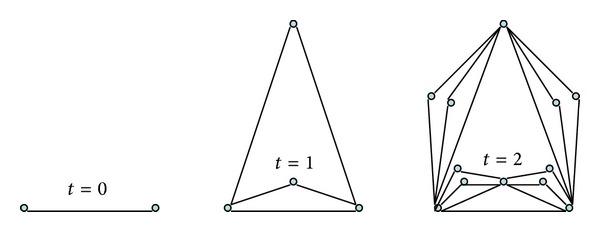
A depiction of graphs *G*(*m*, *t*) produced at iterations *t* = 0, 1, 2 with *m* = 2.

In this section, we introduce the geometric assortative growth model for small-world networks in a deterministic manner, and we denote the network graph by *G*(*m*, *t*) = (*V*(*m*, *t*), *E*(*m*, *t*)) with vertex set *V*(*m*, *t*) and edge set *E*(*m*, *t*) after *t* iteration steps. The construction algorithm of the model is the following: (i) for *t* = 0, *G*(*m*, 0) contains two initial vertices and an edge joining them, namely, *K*_2_; (ii) for *t* ≥ 1, *G*(*m*, *t*) is obtained from *G*(*m*, *t* − 1) by adding *m* new vertices for each edge introduced at step *t* − 1 and attaching them to two end vertices of this edge. As such, we will call an edge a generating edge, if it is used to introduce new vertices in the next iteration step. The first three steps of generation process of the growth model are shown in Figure 1.

In what follows, we will often write *G*(*t*), *V*(*t*), *E*(*t*), and so forth, suppressing the variable *m* if we do not emphasize the specific value of *m*. We denote the two initial vertices in *G*(0) by *v*_0_ and *v*_1_ and the number of new vertices and edges added at step *t* by *L*_*V*_(*t*) and *L*_*E*_(*t*), respectively. Therefore, we have *L*_*V*_(0) = 2 and *L*_*E*_(0) = 1. From the above construction, it is easy to see that *L*_*V*_(*t*) = *mL*_*E*_(*t* − 1) and *L*_*E*_(*t*) = 2*mL*_*E*_(*t* − 1), which give rise to *L*_*E*_(*t*) = (2*m*)^*t*^ and *L*_*V*_(*t*) = *m*(2*m*)^*t*−1^ for any *t* ≥ 1. We have the following result.

**Proposition 1**

The order and size of the graph *G*(*t*) are

(1) $$\begin{array}{c} \left|V\left(t\right)\right|=\frac{m{\left(2m\right)}^{t}+3m-2}{2m-1},\;\;\left|E\left(t\right)\right|=\frac{{\left(2m\right)}^{t+1}-1}{2m-1}, \end{array}$$

respectively. Moreover, the average degree of *G*(*t*) is

(2) $$\begin{array}{c} \bar{\delta }\left(t\right)=4-\frac{12m-6}{m{\left(2m\right)}^{t}+3m-2}. \end{array}$$

**Proof**

They can be directly checked by |*V*(*t*)| = ∑_*i*=0_^*t*^ *L*_*V*_(*i*), |*E*(*t*)| = ∑_*i*=0_^*t*^ *L*_*E*_(*i*), and $\bar{\delta }(t)=2|E(t)|/|V(t)|$.

Note that the average degree tends to 4 as *t* → *∞* irrespective of *m*. This kind of sparse networks are common in both humanly constructed and natural networks [20, 21]. Some more sophisticated properties will be addressed in the following. We will, for example, improve the one-point average-degree characterization of a network by considering assortativity, a two-point correlation quantity.

## 3. Topological Properties of *G*(*m*, *t*)

Thanks to the deterministic nature of the graphs *G*(*m*, *t*), in this section we will derive analytically some main topological properties, namely, the degree distribution, degree correlations, clustering coefficient, transitivity coefficient, and diameter.

### 3.1. Degree Distribution

A fundamental quantity characterizing the structure and driving the behavior of a large network is the probability distribution function *P*(*δ*) of vertex degree *δ*. It is the probability that a randomly chosen vertex has *δ* direct neighbors. It is often convenient to consider the cumulative degree distribution [17, 21, 22]

(3) $$\begin{array}{c} P_{\text{cum}}\left(\delta \right)=\overset{\infty }{\underset{\delta^{\prime }=\delta }{\sum }}P\left(\delta^{\prime }\right), \end{array}$$

which indicates the proportion of the vertices whose degree is greater than or equal to *δ*. An appealing property of the cumulative distribution is: Networks with exponential degree distribution, namely, *P*(*δ*) ~ *e* ^−*αδ*^, also have exponential cumulative distribution with the same exponent. Indeed,

(4) $$\begin{array}{c} P_{\text{cum}}\left(\delta \right)=\overset{\infty }{\underset{\delta^{\prime }=\delta }{\sum }}P\left(\delta^{\prime }\right)\approx \overset{\infty }{\underset{\delta^{\prime }=\delta }{\sum }}e^{-\alpha \delta^{\prime }}=\left(\frac{e^{\alpha }}{e^{\alpha }-1}\right)e^{-\alpha \delta }. \end{array}$$

The Watts-Strogatz small-world model [1] also has an exponential degree distribution as we will study here. We mention that there are some other geometric growth models proposed in the literature, which follow another ubiquitous degree distribution: scale-free distributions; see, for example, [18, 23, 24].

**Proposition 2**

The cumulative degree distribution of *G*(*t*) follows an exponential distribution *P*_*cum*_(*δ*)~(2*m*)^−(*δ*/2*m*)^ for large *t*.

**Proof**

Let *δ*_*v*_(*t*) denote the degree of vertex *v* in *G*(*t*). Let *t*_*i*,*v*_ be the step at which a vertex *v* is added to the graph. From the construction, all the vertices in the graph (except two initial vertices *v*_0_ and *v*_1_) are always connected to two generating edges and will increase their degrees by 2*m* at the next iteration.

At *t* = 0, the graph has two initial vertices *v*_0_ and *v*_1_ with degree 1; that is, *δ*_*v*_0__(0) = *δ*_*v*_1__(0) = 1. For *t* ≥ 1, by construction, we have

(5) $$\begin{array}{c} \delta_{v_{0}}\left(t\right)=\delta_{v_{1}}\left(t\right)=1+m+\left(t-1\right)m^{2}. \end{array}$$

For other vertices, we have *δ*_*v*_(*t*_*i*,*v*_) = 2 and *δ*_*v*_(*t* + 1) = *δ*_*v*_(*t*) + 2*m*. Thus,

(6) $$\begin{array}{c} \delta_{v}\left(t\right)=2\left(m\left(t-t_{i,v}\right)+1\right), \end{array}$$

for *t* ≥ *t*_*i*,*v*_. Hence, the degree distribution of the graph *G*(*t*) is as follows. The number of vertices of degree 2 · 1, 2 · (*m* + 1), 2 · (2*m* + 1),…, 2 · (*m*(*t* − 1) + 1), equals *m*(2*m*)^*t*−1^,  *m*(2*m*)^*t*−2^, *m*(2*m*)^*t*−3^,…, *m*, respectively, and the degrees of two initial vertices are given by (5).

Using (6), we have *P*_cum_(*δ*) = *P*(*t*′ ≤ *τ* = *t* − (*δ* − 2)/2*m*). Thus, by exploiting Proposition 1, we obtain

(7) Pcum(δ)≈∑t′=0τLV(t′)|V(t)|‍=2(2m−1)m(2m)t+3m−2+∑t′=1τm(2m)t′−1(2m−1)m(2m)t+3m−2‍=m(2m)τ+3m−2m(2m)t+3m−2~(2m)−(δ/2m).

for large graphs (i.e., *t* → *∞*).

We will make use of the exact degree distribution of *G*(*t*) obtained in the above proof to study the clustering coefficient in the sequel.

### 3.2. Degree Correlations (Average Neighbor Degree)

To uncover correlations between the degrees of connected vertices, the average neighbor degree, *k*_*nn*_(*δ*), for vertices of degree *δ*, is defined as the average degree of nearest neighbors of vertices with degree *δ* as a function of this degree value [25, 26]. If *k*_*nn*_(*δ*) is an increasing function of *δ*, vertices with high-degree have a larger probability to be connected with large degree vertices. In this case, the graph is said to be assortative and this property is referred to in social sciences as assortative mixing [12]. Generally, assortativity is the tendency of entities to seek out and group with those other entities that exhibit similar characteristics. In contrast, a decreasing behavior of *k*_*nn*_(*δ*) defines a disassortative graph, in the sense that high-degree vertices have a majority of neighbors with low-degree, whereas the opposite holds for low-degree vertices. In the absence of degree correlations, *k*_*nn*_(*δ*) is a constant. We remark here that the concept of *k*_*nn*_(*δ*) is related to the groupie in graphs (see, e.g., [27, 28]).

**Proposition 3**

The average neighbor degree for *G*(*t*) is, respectively,

(i)

(8) $$\begin{array}{c} k_{nn}\left(\delta_{0}\right)=\frac{t\delta_{0}+mt+2m^{2}t+2m-t}{1+m+\left(t-1\right)m^{2}}, \end{array}$$

where *δ*_0_ = 1 + *m* + (*t* − 1)*m* ^2^ (cf. (5)) is the degree of two initial vertices *v*_0_ and *v*_1_:

(ii)

(9) knn(δ)=m2δ2(2m−1)+m(2m)3(2m−1)2δ+m22m−1−m(t+3)(2m)(1+(δ/2))/m(2m)tδ,

where *δ* = 2(*m*(*t* − *t*_*i*_) + 1) (cf. (6)) is the degree of other vertices added to the network at step *t*_*i*_ ≥ 1.

**Proof**

We first show (9). It is clear that all vertices introduced at the same iteration step have the same degree. No vertices (except *v*_0_ and *v*_1_) added to the network at the same step will be connected to each other. When a new vertex is added to the network, it connects vertices with larger degrees and it will connect vertices with smaller degrees in the subsequent steps. From (6), for vertices introduced to the network at step *t*_*i*_ ≥ 1, they have the same degree *δ* = 2(*m*(*t* − *t*_*i*_) + 1).

Let *δ*(*t*_*i*_, *t*) represent the degree at step *t* of a vertex that was generated at step *t*_*i*_. Thus, *δ*(*t*_*i*_, *t*) = 2(*m*(*t* − *t*_*i*_) + 1). We have

(10) knn(δ)=1LV(ti)δ(ti,t)×(2m∑ti′=1ti−1LV(ti′)δ(ti′,t)+2m∑ti′=ti+1tLV(ti′)δ(ti′,t).+LV(0)δ(0,t)).

The first sum on the left-hand side of (10) accounts for the adjacencies made to vertices with larger degree; namely, 1 ≤ *t*_*i*_′ < *t*_*i*_, and the second sum represents the edges introduced to vertices with a smaller degree at each step *t*_*i*_′ > *t*_*i*_. The last term in (10) accounts for the adjacencies made to the initial vertices *v*_0_ and *v*_1_.

From (10), we derive that

(11) knn(δ)=1(2m)ti(m(t−ti)+1)×(2m(2m+2m2t)((2m)ti−1−1)2m−1−4m3((2m)ti−1−1+2m(ti−1))2m−1+4m3((2m)ti−1−1)(2m−1)2+(2m)ti+1(2m+2m2t)((2m)t−ti−1)2m−1−((4m3((ti+1)(2m)ti((2m)t−ti−1)+(2m)t(t−ti)))×(2m−1)−1)+4m3(2m)ti+1((2m)t−ti−1)(2m−1)2+2(1+m+(t−1)m2)).

Feed *δ* = 2(*m*(*t* − *t*_*i*_) + 1) into the above expression, eliminate *t*_*i*_, and simplify the consequential expression giving rise to (9) finally.

Next, for the two initial vertices with degree *δ*_0_ = 1 + *m* + (*t* − 1)*m* ^2^, we obtain

(12) $$\begin{array}{c} k_{nn}\left(\delta_{0}\right)=\frac{m}{\delta_{0}}\overset{t}{\underset{t_{i}^{\prime }=0}{\sum }}\delta \left(t_{i}^{\prime },t\right)‍=\frac{2m\left(t+1\right)+m^{2}t\left(t+1\right)}{1+m+\left(t-1\right)m^{2}}, \end{array}$$

which yields to (8) as desired.

Note that, as *t* tends to infinity, (8) is tantamount to (*δ*_0_ + *m* + 2*m* ^2^ − 1)/*m* ^2^ and the last term on the right-hand side of (9) is vanishing. Therefore, we conclude that *k*_*nn*_(*δ*) is approximately a linear function of *δ* for large *t*, which implies that our model *G*(*t*) undergoes assortative growth.

To find the impact of parameter *m*, we note that (8) decreases with *m*, while (9) increases with *m* for large *t*. Since the contribution to the degree correlation of the two initial vertices of *G*(*t*) is small, we can safely think of *k*_*nn*_(*δ*) as an increasing function with respect to *m* for large graphs, meaning that *G*(*m*, *t*) shows more significant assortative mixing for larger *m*. This fact will be even clearer drawing on the correlation coefficient (see below).

### 3.3. Degree Correlations (Pearson Correlation Coefficient)

Another quantity often used to probe the assortativity is the Pearson correlation coefficient *r* of vertices connected by an edge [12, 13],

(13) $$\begin{array}{c} r=\frac{\left|E\right|\sum_{i}^{}j_{i}k_{i}‍-{\left(\sum_{i}^{}\left(1/2\right)\left(j_{i}+k_{i}\right)\right)}^{2}}{\left|E\right|\sum_{i}^{}\left(1/2\right)\left(j_{i}^{2}+k_{i}^{2}\right)‍-{\left(\sum_{i}^{}\left(1/2\right)\left(j_{i}+k_{i}\right)\right)}^{2}}, \end{array}$$

where *E* is the edge set of the graph in question and *j*_*i*_ and *k*_*i*_ are the degrees of the vertices at the ends of the *i*th edge, with *i* = 1,2,…, |*E*|. It lies in the range −1 ≤ *r* ≤ 1. This coefficient is zero for uncorrelated graph and positive or negative for assortative or disassortative mixing, respectively. Let *r*(*t*) be the degree-degree Pearson correlation coefficient of *G*(*t*). We have the following result.

**Proposition 4**

The Pearson correlation coefficient of *G*(*t*) is

(14) r(t)=(1+o(1))((((2m)2+1)/(2m−1))(2m)5+2t)−((9/(4(2m−1)2))(2m)6+2t)(((3(2m)2+1)/(2(2m−1)))(2m)5+2t)−((9/(4(2m−1)2))(2m)6+2t)⟶2(2m)2+2−(9/(2m−1))3(2m)2+1−(9/(2m−1)),

as *t* → *∞*.

It is direct to check that (10) is positive for all *m* ≥ 1. It is an increasing function with *m* and has upper bound 2/3. Therefore, for large *t*, the growth model *G*(*m*, *t*) is assortative for all *m* ≥ 1 and the level of assortativity increases with *m*. This also justifies the above discussion of assortativity based on local quantity *k*_*nn*_(*δ*).

**Proof**

Following the notation in [14], we denote by 〈*j*_*i*_, *k*_*i*_〉 the *i*th edge in *G*(*t*) connecting two vertices with degree *j*_*i*_ and *k*_*i*_, respectively. By (5), the edge in *G*(0) is thus 〈1 + *m* + (*t* − 1)*m* ^2^, 1 + *m* + (*t* − 1)*m* ^2^〉. (2*m*)^*t*_*i*_^ new edges are added to the network at iteration step *t*_*i*_ ≥ 1. These edges will connect new vertices to every vertex in *G*(*t*_*i*_ − 1), whose degree distribution at *t*_*i*_ − 1 is *δ*(*l*, *t*_*i*_ − 1) = 2(*m*(*t*_*i*_ − 1 − *l*) + 1) for 1 ≤ *l* ≤ *t*_*i*_ − 1, and *δ*(0, *t*_*i*_ − 1) = 1 + *m* + (*t*_*i*_ − 2)*m* ^2^. Here, the *δ* notation is defined in the proof of Proposition 3.

At each of the subsequent steps of *t*_*i*_ − 1, the degrees of all these vertices will gain 2*m* except *v*_0_ and *v*_1_, whose degrees will gain *m*. Consequently, at iteration step *t* ≥ *t*_*i*_, the number of edges 〈2*m*(*t* − *t*_*i*_) + 2,2(*m*(*t*_*i*_ − *l*) + 1)〉 for 1 ≤ *l* ≤ *t*_*i*_ − 1 is (2*m*)^*l*^, and the number of edges 〈2*m*(*t* − *t*_*i*_) + 2,1 + *m* + (*t* − 1)*m* ^2^〉 is 2*m*.

We now can evaluate these sums in (13) for large *t*,

(15) ∑i=1|E(t)|jiki‍=(1+m+(t−1)m2)2+2m(1+m+(t−1)m2)×∑ti=1t(2m(t−ti)+2)‍+2∑ti=2t ∑l=1ti−1‍(2m(t−ti)+2)×(m(ti−1)+1)(2m)l=((2m)2+1)(2m)4+t+o((2m)t).

Likewise, we have

(16) ∑i=1|E(t)|(ji+ki)‍ =2(1+m+(t−1)m2)2  +∑ti=1t(2m(1+m+(t−1)m2)+2m(t−ti)+2)‍  +∑ti=2t ∑l=1ti−1(2(m(ti−l)+1)+2m(t−ti)+2)(2m)l‍ =32m−1(2m)3+t+o((2m)t),∑i=1|E(t)|(ji2+ki2)‍=(3(2m)2+1)(2m)4+t+o((2m)t).

Feeding these quantities into the definition (13), we then arrive at the desired result.

### 3.4. Clustering Coefficient

The clustering coefficient [1] is a good indicator of local clustering, namely, the local density of triangles, and thus often used to characterize small-world networks. In a network *G* = (*V*, *E*), the clustering coefficient *c*(*v*) of a vertex *v* ∈ *V* is the ratio of the total number *e*_*v*_ of edges that actually exist between all its *δ*_*v*_ nearest neighbors and the number *δ*_*v*_(*δ*_*v*_ − 1)/2 of all possible edges between them. More precisely,

(17) $$\begin{array}{c} c\left(v\right)=\frac{2e_{v}}{\delta_{v}\left(\delta_{v}-1\right)}. \end{array}$$

The clustering coefficient *c*(*G*) of the whole network *G* is the average of all individual *c*(*v*)'s,

(18) $$\begin{array}{c} c\left(G\right)=\frac{\sum_{v\in V}^{}c\left(v\right)}{\left|V\right|}. \end{array}$$

In what follows, we compute the clustering coefficient for the growth model *G*(*t*).

**Proposition 5**

The clustering coefficient of *G*(*t*) is

(19) c(G(t)):=c(t)=2m−1m(2m)t+3m−2×((2m)t−1+(1/m)ln⁡⁡(2m)−12mΦ(2m,1,t+1m),+41+m+(t−1)m2),

where the function Φ represents the Lerch transcendent (see [29, Section 1.11]).

**Proof**

When a new vertex *v* is added to the graph, it is easy to see *δ*_*v*_ = 2 and *e*_*v*_ = 1. Furthermore, every subsequent addition of an edge attached to this vertex will increase both parameters by one unit. Therefore, we have *e*_*v*_ = *δ*_*v*_ − 1 for every vertex at every step. Thus,

(20) $$\begin{array}{c} c\left(v\right)=\frac{2e_{v}}{\delta_{v}\left(\delta_{v}-1\right)}=\frac{2}{\delta_{v}}. \end{array}$$

Drawing on this relationship, the degree distribution obtained in Proposition 2 can be useful for calculation of the clustering coefficient of *G*(*t*).

Indeed, the number of vertices with clustering coefficient 1, 1/(*m* + 1), 1/(2*m* + 1),…, 1/(*m*(*t* − 1) + 1), 2/(1 + *m* + (*t* − 1)*m* ^2^), equals, respectively, *m*(2*m*)^*t*−1^, *m*(2*m*)^*t*−2^, *m*(2*m*)^*t*−3^,…, *m*, 2. Consequently, we obtain

(21) c(G(t))=1|V(t)|×(∑i=1t1(i−1)m+1·m(2m)t−i‍+41+m+(t−1)m2)=2m−1m(2m)t+3m−2×((2m)t−1+(1/m)ln⁡⁡(2m)−12mΦ(2m,1,t+1m)+41+m+(t−1)m2),

as desired.

For large graphs (i.e., *t* → *∞*), the right-hand side of (19) approaches

(22) $$\begin{array}{c} \frac{\left(2m-1\right){\left(2m\right)}^{1/m}}{2m^{2}}\ln\left(2m\right), \end{array}$$

which is a decreasing function with respect to *m*. Hence, for larger *m*, the level of local clustering becomes lower eventually. This is not quite surprising since a large bunch of vertices will be added to the network at each iteration when *m* and *t* become large, which mitigate the coefficient.

### 3.5. Transitivity Coefficient

Transitivity is an important property especially in the analysis of social networks; see for example [21, 30, 31]. Let *T*(*G*) be the number of triangles and *Q*(*G*) be the number of paths of length two in a graph *G*. Then the transitivity coefficient *c*′(*G*) of *G* can be defined as

(23) $$\begin{array}{c} c^{\prime }\left(G\right)=\frac{3T\left(G\right)}{Q\left(G\right)}. \end{array}$$

A brief discussion of the relationship between clustering and transitivity coefficients can be found, for example, in [14].

**Proposition 6**

The transitivity coefficient of *G*(*t*) is

(24) c′(G(t)):=c′(t)=(1+o(1))3m3((2m)t−1)(2m−1)((2m)2+1)(2m)t+1⟶3m22(2m−1)((2m)2+1),

as *t* → *∞*.

**Proof**

We first calculate *T*(*G*(*t*)). Note that, if the number of generating edges after iteration *t* − 1 is *a*, the number of new triangles introduced to the graph after iteration *t* is 3*a*. Since *a* = *L*_*E*_(*t* − 1), we obtain

(25) T(G(t))=T(G(t−1))+mLE(t−1)=T(G(t−1))+m(2m)t−1,

which together with the initial value *T*(*G*(1)) = *m* gives

(26) $$\begin{array}{c} T\left(G\left(t\right)\right)=\frac{m\left({\left(2m\right)}^{t}-1\right)}{2m-1}, \end{array}$$

for *t* ≥ 1.

The number of paths of length two, *Q*(*G*(*t*)), can be derived as follows by using the degree distribution again:

(27) Q(G(t))=m(2m)t−1+m∑k=1t−1(2(km+1)2)(2m)t−k−1+2(1+m+(t−1)m22)=(2m)2+1m2(2m)t+1+o((2m)t),

which, along with (26), leads to the stated result.

Clearly, the left-hand side of (24) is a decreasing function of *m*. Recalling the comments after Proposition 5, we see that the difference between clustering and transitivity coefficients of *G*(*t*) is by and large quantitative. This is because they measure a quite similar property of networks.

### 3.6. Diameter

Network diameter, namely, the largest length of the shortest paths between all pairs of vertices, is a measure of the transmission performance and communication efficiency. We show analytically the diameter of our growth model and find a quantitative difference between *m* = 1 and *m* > 1.

**Proposition 7**

The diameter diam⁡(*G*(*m*, *t*))∶ = diam⁡(*t*) of *G*(*m*, *t*) equals *t* for *m* = 1 and *t* + 1 for *m* ≥ 2.

**Proof**

The case of *m* = 1 was shown in [14]. In what follows, we take over their method to study *m* ≥ 2.

Clearly, diam⁡(*G*(*m*, 0)) = 1 and diam⁡(*G*(*m*, 1)) = 2. At each step *t* ≥ 2, the longest distance between two vertices is for some vertices added at this step corresponding to different generating edges at the last step. Consider two vertices introduced at step *t* ≥ 2 corresponding to different generating edges, say *u*_*t*_ and *v*_*t*_. The vertex *u*_*t*_ is adjacent to two vertices, and one of them must have been added to the graph at step *t* − 2 or earlier.

If *t* = 2*k* is even, *u*_*t*_ can reach some vertex in *G*(*m*, 0) by *k* jumps, and the same thing is true for vertex *v*_*t*_. Therefore, diam⁡(*G*(*m*, 2*k*)) ≤ 2*k* + 1. If *t* = 2*k* + 1 is odd, *u*_*t*_ can reach some vertex in *G*(*m*, 1) by *k* jumps, and the same thing is true for vertex *v*_*t*_. Therefore, diam⁡(*G*(*m*, 2*k* + 1)) ≤ 2*k* + 2. These bounds are attained by pairs of vertices *u*_*t*_ and *v*_*t*_ created at iteration *t*, which correspond to different generating edges and have the property of being connected to two vertices introduced at steps *t* − 1 and *t* − 2, respectively. Consequently, we have diam⁡(*G*(*m*, *t*)) = *t* + 1 for all *m* ≥ 2 and *t* ≥ 1.

From Proposition 1, we have, for *t* large,

(28) $$\begin{array}{c} \ln\left|V\left(t\right)\right|=\ln\left(\frac{m{\left(2m\right)}^{t}+3m-2}{2m-1}\right)\sim t\ln\left(2m\right). \end{array}$$

Hence, we obtain the logarithmic scale

(29) $$\begin{array}{c} \mathrm{diam}\left(t\right)\sim \frac{\ln\left|V\left(t\right)\right|}{\ln\left(2m\right)}, \end{array}$$

which together with high clustering (Propositions 5 and 6) justifies the small-world characteristics [1] of our growth model.

## 4. Conclusion

We have studied a geometric assortative growth model *G*(*m*, *t*) for small-world networks in a deterministic way. We obtain analytical solutions of main properties of the model, such as the degree distribution and correlations, clustering and transitivity coefficients, and graph diameter, in the full spectrum of parameter *m*. The *G*(*m*, *t*) model holds both tunable small-world and tunable assortative mixing behaviors. This should be useful to guide the research and development of varied social networks. On the other hand, the deterministic character of this graph family should facilitate the exact calculation of other network-oriented quantities, including average path length, hyperbolicity [32], modular structure, and motifs [33].

The introduction of tunable parameter *m* also brings a range of open questions for future research. In addition to those mentioned before, here are more examples: how can we make a trade-off between local clustering and assortativity by tuning *m* since they have opposite monotonicity? What if *m* = *m*(*t*) is a function of time?

## Figures and Tables

**Table 1:** Properties of model *G*(*m*, *t*).

	*m* = 1^a^	*m* ≥ 2
Cumulative degree distribution *P*_cum_(δ)	(2*m*)^−δ/2*m*^ for large *t*	
Average neighbor degree *k*_*nn*_(δ)	$\frac{m^{2}\delta }{2(2m-1)}$ for large *t*	
Pearson correlation coefficient *r*(*t*)	Increase with *m* as $\frac{2{\left(2m\right)}^{2}+2-\left(9/\left(2m-1\right)\right)}{3{\left(2m\right)}^{2}+1-\left(9/\left(2m-1\right)\right)}$ for large *t*	
Clustering coefficient *c*(*t*)	Decrease with *m* as $\frac{\left(2m-1\right){\left(2m\right)}^{1/m}}{2m^{2}}\ln\left(2m\right)$ for large *t*	
Transitivity coefficient *c*′(*t*)	Decrease with *m* as $\frac{3m^{2}}{2\left(2m-1\right)\left({\left(2m\right)}^{2}+1\right)}$ for large *t*	
Diameter diam⁡(*t*)	*t*	*t* + 1

^a^: the properties for *G*(1, *t*) were obtained in [14].
